# The Influence of Pain Distribution on Walking Velocity and Horizontal Ground Reaction Forces in Patients with Low Back Pain

**DOI:** 10.1155/2012/214980

**Published:** 2012-04-04

**Authors:** Maureen J. Simmonds, C. Ellen Lee, Bruce R. Etnyre, G. Stephen Morris

**Affiliations:** ^1^University of Texas, Health Sciences Center, San Antonio, TX 78229, USA; ^2^A. T. Still Research Institute, A. T. Still University, Missouri Campus, 800 W Jefferson Street, Kirksville, MO 63501, USA; ^3^Department of Physical Therapy, University of Manitoba, Winnipeg, MB, Canada R3T 2N2; ^4^Department of Kinesiology, Rice University, Houston, TX 77251-1892, USA; ^5^Department of Rehabilitation Services, St. Jude Children's Research Hospital, Memphis, TN 38105, USA

## Abstract

*Objective*. The primary purpose of this paper was to evaluate the influence of pain distribution on gait characteristics in subjects with low back problems (LBP) during walking at preferred and fastest speeds. *Design*. Cross-sectional, observational study. *Setting*. Gait analysis laboratory in a health professions university. *Participants*. A convenience age- and gender-matched sample of 20 subjects with back pain only (BPO), 20 with referred leg pain due to back problems (LGP), and 20 pain-free individuals (CON). *Methods and Measures*. Subjects completed standardized self-reports on pain and disability and were videotaped as they walked at their preferred and fastest speeds along a walkway embedded with a force plate. Temporal and spatial gait characteristics were measured at the midsection of the walkway, and peak medial, lateral, anterior, and posterior components of horizontal ground reaction forces (hGRFs) were measured during the stance phase. *Results*. Patients with leg pain had higher levels of pain intensity and affect compared to those with back pain only (*t* = 4.91, *P* < .001 and *t* = 5.80, *P* < 0.001, resp.) and walking had an analgesic effect in the BPO group. Gait velocity was highest in the control group followed by the BPO and LGP group and differed between groups at both walking speeds (*F*_2.57_ = 13.62, *P* < .001 and *F*_2.57_ = 9.09, *P* < .001, for preferred and fastest speed condition, resp.). When normalized against gait velocity, the LGP group generated significantly less lateral force at the fastest walking speed (*P* = .005) and significantly less posterior force at both walking speeds (*P* ≤ .01) compared to the control group. *Conclusions*. Pain intensity and distribution differentially influence gait velocity and hGRFs during gait. Those with referred leg pain tend to utilize significantly altered gait strategies that are more apparent at faster walking speeds.

## 1. Introduction

Low back pain (LBP) remains a prevalent and persistent problem that frequently compromises physical function, including walking. Best evidence management for LBP now emphasizes remaining active or resuming or increasing usual activity as soon as possible [1–3], and walking is commonly recommended as a therapeutic exercise [1–5]. Unfortunately, although some people with LBP will remain active, others have difficulty doing so for a variety of physical, psychological and social reasons [5], and this can contribute to the individual's distress and disability and the economic cost of chronic LBP [6]. Pain appears to be a unique domain as a cause of disablement, independent of physical impairment [7]. Given the fundamental nature of walking and the fact that it is an oft prescribed activity for patients with LBP, it is clearly important to have a better understanding of the effect of pain on walking.

Walking is a complex dynamic task that is fundamental to function and that requires an individual to generate and withstand a variety of multidirectional forces around each joint and with the ground, that is, ground reaction forces (GRFs). To date, there is limited knowledge about the gait characteristics of people with chronic LBP. It is known that individuals with low back pain tend to walk slower, and generally take shorter steps that may be asymmetric compared to their age-matched cohorts [8–15]. It was originally believed that this gait strategy was simply due to people with LBP attempting to attenuate the magnitude of internal and external forces exerted or imposed on the body during walking, particularly at heel strike. This was a plausible if untested idea, given that there is a linear increase in vertical GRFs with increased gait velocity, at least in pain-free individuals [9]. However, individuals with pain and other lower limb impairments appear to adopt a variety of alternative strategies during walking, some of which attenuate measured forces and others that do not. Understanding the links between pain and movement is compounded by kinematic variability that makes it difficult to identify “normal” movement and thus what aspect of the individual person's movement pattern may have changed due to pain or injury as opposed to what is usual for them.

We have previously demonstrated that self-report of disability and several gait characteristics are differentially influenced by pain distribution, by walking speed (preferred versus fastest speed) [8], and by walking condition (over ground or on a treadmill) [16]. Treadmills are often used to assess gait but people with or without pain walk differently on a treadmill compared to over ground walking [16]. In another study [8], we showed that individuals with LBP and leg pain (LGP) prefer to walk more slowly compared to those with back pain only (BPO) or with an age- and gender-matched cohort, respectively. However, when challenged to walk as fast as possible, people with BPO are able to walk as fast and withstand comparable vertical GRFs as a pain-free control group. This was not the case for people with referred leg pain. People with LBP and leg pain were not able to walk as fast as those with BPO and were clearly outperformed by pain-free subjects. Finally, we found that individuals with leg pain attenuated vertical GRFs not only by reducing the velocity of walking, but also through an asymmetrical gait strategy that included prolonged loading and push-off response phases that effectively reduced the rate at which the vertical GRFs were imposed.

Given that pain distribution has a differential effect on walking and on vertical GRFs, it is plausible that horizontal GRF components may also be similarly affected. Horizontal GRFs generated during walking consist of anteroposterior (AP) and mediolateral (ML) force components. AP forces are measures of braking-driving forces whereas ML forces are indicators of mediolateral motion during gait.

In regards to AP forces, it is generally assumed that people with BPO and especially those with referred leg pain from their LBP will withstand less braking force and potentially exert less driving force, however, this has not been well established. It is known that following total knee arthroplasty, people withstand similar braking forces but generate less driving force than their pain-free counterparts [17]. However, the mechanisms, the robustness, and the implications of this finding are not clear and it is not known whether this gait characteristic is similar in those with back problems, with or without leg pain.

ML forces are measures of lateral limb displacement, which have received even less attention to date. Although it has been suggested that ML forces may be used as a proxy measure for postural control in the frontal plane during walking [9, 10], there is little evidence for this. Previous studies have found that people with LBP have greater postural sway during sitting and standing tasks, however, the greater postural sway occurs in all directions as compared to pain-free individuals [12–15, 18]. It is not clear whether the observed increase in postural sway is due to compromised postural mechanisms or simply a way of attenuating or alternating loads on sensitive structures in the back. In our laboratory, we have shown that for people with LBP performing a sit to stand or reach forward task, a much greater lateral displacement of center of pressure occurs during task performance compared to that of pain-free individuals [15, 18]. However patients with LBP also perform tasks more slowly and this in itself increases the challenge to balance and postural control. Albeit slow movements do attenuate the rate of change in the loading forces that must be tolerated. To date it is not clear whether the observed differences in movement patterns or potential postural control problems of patients with back pain are causes, correlates, or consequences of LBP. For example, several lines of research have suggested that movement compromise is not simply a direct consequence of pain but rather—and at least in part—evidence of the motor expression of a more systemic problem. For example, generalized psychomotor slowing occurs with health problems that are not necessarily painful. For example, some cancers, HIV, chronic fatigue syndrome, and mental health disorders [19]. On the other hand, experienced and anticipated pain and experimentally induced pain influence movement and activity [20]. Thus, although it is known that pain is associated with generalized psychomotor slowing and movement variability, the specific movement characteristics of people with pain are neither well described nor well understood. An integrated understanding of pain and GRF characteristics during walking under different speed conditions in people with LBP is important. Such an understanding could ultimately assist clinicians to assess and manage walking exercises more appropriately.

 Thus, the primary purpose of this study was to evaluate the influence of pain distribution on hGRFs during gait in people with low back problems (LBP) walking at preferred and fastest speeds. These two walking speeds reflect conditions that individuals are likely to face during community ambulation. Gait velocity was controlled as a covariate since GRF components usually increase with gait velocity [21].

Given that gait velocity is a reasonable indicator of walking capacity and overall function, a secondary aim was to better understand the predictors of gait velocity at preferred and fastest speed in people with LBP with and without referred leg pain.

It was hypothesized that: (1) the BPO group and the control group would generate similar peak medial, lateral, anterior, and posterior forces at either walking speed; (2) the LGP group would generate significantly less peak medial, lateral, anterior and peak posterior forces than the control group or the BPO group at both walking speeds; (3) that pain and perceived walking ability would be significant predictors of gait velocity under both walking speed conditions.

## 2. Methods

The study was part of a comprehensive gait study that involved kinetic, kinematic and electromyographic analyses. This paper limits its scope to a description of horizontal GRFs during gait, controlling for gait velocity, and identifying predictors of gait velocity. The study was approved by the Institutional Review Board of Texas Woman's University.

### 2.1. Subjects

Subjects with low back problems were recruited from an outpatient orthopedic spine clinic in a large urban city. Subjects who met the criteria were provided with information about the study purpose and procedures and given an opportunity to ask questions. Those who agreed to participate then signed an informed consent and an appointment was made for them to attend the university located gait lab where the testing took place.

Subjects were assigned to one of two patient groups (*n* = 20 per group) based on their distribution of pain. Subjects were included if they were between 18 to 65 years old, had a current episode of recurrent LBP and/or unilateral referred leg pain from the lower back (as determined by the orthopedic spine specialist) and were under current medical care. Exclusion criteria for all groups were grade 3 obesity (body mass index greater than 41 kg/m^2^) [22], true leg length discrepancy greater than 2 cm [23–26], current systemic or musculoskeletal conditions other than LBP or unilateral referred leg pain, a history of idiopathic scoliosis, spondylolisthesis, ankylosing spondylosis, spine fusion surgery, or any lower extremity orthopedic surgery within one year of potential study participation. For participants in the LGP group, data was recorded from the painful side. And the tested lower limb of participants of the control and BPO groups was matched to the same side.

The control subjects were matched by gender and age (±5 years) and comprised 20 pain-free individuals who had not experienced LBP within the previous 12 months that had required medical attention.

### 2.2. Self-Report Measures

Subjects with low back problems completed the following standardized self-report measures of pain and disability.

#### 2.2.1. Pain

 Subjects were asked to complete a pain drawing that showed the distribution of their pain. Pain that was distributed in the lumbar area or buttock(s) was classified as back pain and subjects were assigned to the BPO group. Pain that was distributed inferiorly to the gluteal fold was deemed referred pain and subjects were assigned to the LGP group.

Two visual analogue scales were used to measure pain intensity and pain affect. Anchor words for pain intensity were “no pain” and “most severe pain imaginable”. Anchor words for pain affect were “pain doesn't bother me” and “pain couldn't bother me more.”

#### 2.2.2. Disability

Disability was measured using the Roland and Morris Disability Questionnaire (RMDQ) [27]. The RMDQ is a 24-item measure of current activity limitation from LBP. The total RMDQ is a sum of activities that are limited, with higher total scores representing greater activity limitation.

#### 2.2.3. Fear-Avoidance—Physical Activity

The *Fear-Avoidance Beliefs Questionnaire *(FABQ) was originally developed by Waddell et al. [28] to assess fear avoidance beliefs in patients with back pain. The original version of the FABQ contained 16 items divided into two subscales: fear-avoidance beliefs about physical activity (5 items) and fear avoidance beliefs about work (11 items). Each item is rated on a seven point Likert Scale (“do not agree at all” = 0 to “completely agree” = 6). Higher scores reflect greater levels of fear-avoidance. The scale has moderate internal consistency [29]. In the current study, the FABQ-physical activity subscale was used and subjects were requested to respond to each item as it applied to walking.

#### 2.2.4. Pain Behavior Checklist—Distorted Ambulation

The Pain Behavior Check List (PBCL) is a widely used measure of pain behavior. The PBCL assesses four categories of pain behaviors (distorted ambulation, affective distress, facial/audible expressions, and help seeking behavior). Each item is rated on a seven point Likert Scale (“never” = 0 to “Very often” = 6). Higher scores reflect greater levels of pain behaviors. In the current study, only the distorted ambulation subscale was used.

### 2.3. Equipment

All participants walked on an elevated walkway (0.10 m × 9.07 m × 1.22 m) with a force platform (Advanced Medical Technology Inc.) (AMTI) Model OR6-7-2000) embedded at its midpoint. The AMTI force platform has good reliability with very low crosstalk between vertical and horizontal forces (*F*_*y*_ = 0.1–0.4%; *F*_*x*_ = 0.2–0.3%) and minimal mean errors (*F*_*z*_ = 1.3–2.8%) [30]. The force platform was located at midpoint of the walkway to ensure constant velocity was achieved. Pilot data showed that participants were able to maintain constant gait velocity across the middle section of the walkway.

Signals from the force platform were low-pass filtered (*f*_*c*_ = 1050 Hz) and amplified (gain = 4000) through a six-channel strain gauge AMTI amplifier, and interfaced with the Ariel Performance Analysis System (APAS) (Ariel Dynamics, Inc) via an analog-to-digital interface board (sampling at 1000 Hz and 16-bit resolution). Two-seconds of GRF data during each static standing and walking trial were exported at 1/1000th-second intervals to a Microsoft Excel spreadsheet. Body weight was calculated by averaging two seconds vertical GRF data during the static standing trial. And peak hGRF parameters in anterior, posterior, medial, and lateral directions were obtained from the Excel file and used in the subsequent analyses. Gait velocity was normalized using the following calculation ((stride length/body height)/stride time).

### 2.4. Procedures

All subjects signed an institutionally approved informed consent and video release prior to participation. Demographic and self-reported anthropometric data were collected from all subjects, and an assessment was carried out to ensure that there was no leg length discrepancy [31]. Individuals with LBP then addressed questions related to their LBP history and completed the self-report pain and disability questionnaires. Reflective 1 cm spherical markers were next applied bilaterally to the posterior aspect of calcanei in order to obtain kinematic data on temporal and spatial gait characteristics. Finally, body weight was obtained from the force platform during a static standing trial. For all walking trials, participants walked a marked distance of 7.62 m on the walkway at two self-selected walking speed conditions: preferred and fastest. The order of the walking speed conditions were counterbalanced by alternating their order within each group. One practice trial was performed prior to the three test trials that were recorded for each condition. A one-minute standing rest occurred between each test trial. Finally, posttest assessments of pain intensity and pain affect were obtained in the BPO and LGP groups using VASs.

### 2.5. Data Analysis

PASW 18.0.0 statistical software was used for all analyses. Descriptive statistics (mean, standard deviation, minima and maxima) were calculated on all dependent variables. Multivariate analysis of covariance (MANCOVA) with normalized gait velocity as the covariate, was used to compare the horizontal GRF components (peak medial, lateral, anterior and posterior forces) among the three groups of participants during both the preferred and fastest walking speed condition. The alpha level was set at 0.05. In *post hoc* group comparison analyses, the alpha level was set at 0.017 according to the Bonferroni adjustment for the three groups. Paired *t*-tests were used to examine for significant change in pre- to post-pain intensity and pain affect. Finally, Pearson's correlation coefficients were used to evaluate associations between pain and disability measures and gait velocity. And stepwise multiple linear regression analysis was used to identify predictors of preferred and fastest gait velocity in the LBP groups.

## 3. Results

The assumptions of MANCOVA were confirmed. Normalized gait velocity had a significant linear relationship with all horizontal GRF at both walking speeds (*r* = 0.33–0.69, *P* ≤ 0.009). The assumption of homogeneity of regression was met—there was no significant interaction between the normalized gait velocity and the study groups at both the preferred walking speed (*P* = 0.499) and the fastest walking speed (*P* = 0.237). The overall power for the horizontal GRF components analysis during the preferred walking speed was 82% with an effect size of 0.14 and that of the fastest walking speed condition was 88% with an effect size of 0.21.

Descriptive statistics of the demographics of the matched three groups of subjects are presented in Table 1, and descriptive statistics on the low back problems and self-reports of the two LBP groups are presented in Table 2.

### 3.1. LBP History

The long duration of LBP history and even the current episode of LBP (mean 12 and 18 months in LGP and BPO, resp.) is clearly evident. However, perhaps more remarkable is the fact that the self-reported duration of the “current episode of LBP” ranged up to 10 years for those with BPO and 5 years for those with LGP.

### 3.2. Pain

 Mean levels of back pain intensity between the BPO and LGP groups (3.9 ± 2.1 and 5.1 ± 2.2, resp.) at baseline were not significantly different (*t*_1,38_ = −1.87, *P* = 0.07) however pain affect was higher (4.4 ± 2.4 and 6.0 + 2.4, *t*_1,38_ = −2.07, *P* < 0.05, resp.) in the LGP group. Furthermore, a significant change in pre-posttest pain intensity and affect occurred but only in the BPO group. It is noteworthy that this change was a significant reduction in both posttest pain intensity and affect compared to Pretest pain levels.

### 3.3. Disability

There was a trend towards higher mean values of disability (LGP 12.50 ± 4.32, BPO 9.45 ± 5.93, *P* = 0.06), fear avoidance (LGP 10.60 ± 7.42, BPO 6.50 ± 6.02, *P* = 0.07), and distorted ambulation (LGP 12.85 ± 7.04, BPO 9.60 ± 7.80, *P* = 0.18) in the LGP group on all self-report measures but the differences were not statistically significant.

### 3.4. Gait Velocity

Descriptive statistics of the normalized and absolute gait velocity of the three groups are presented in Table 3. In general and terms of mean gait velocity, the LGP were generally outperformed by the BPO and control groups, respectively; however, not all mean differences were significant between groups. Specifically, the BPO group walked significantly slower than the control group at their preferred walking speed (*F*_1.40_, 26.65 = 12.57, *P* ≤ 0.001) but both BPO and control groups had comparable normalized gait velocity during the fastest walking speed condition (*F*_1.79_, 34.05 = 8.82, *P* = 0.175). The LGP group walked significantly slower than the control group at both preferred walking speed (*F*_1.40_ = −12.57, *P* < 0.005) and fastest walking speed conditions (*F*_1.79_, 34.05 = 8.82, *P* < 0.005). The LGP group had comparable normalized gait velocity to the BPO group at preferred speed, but walked significantly slower than the BPO group at fastest speed (*P* = 0.013).

### 3.5. Horizontal Ground Reaction Forces

**Figure 1:**
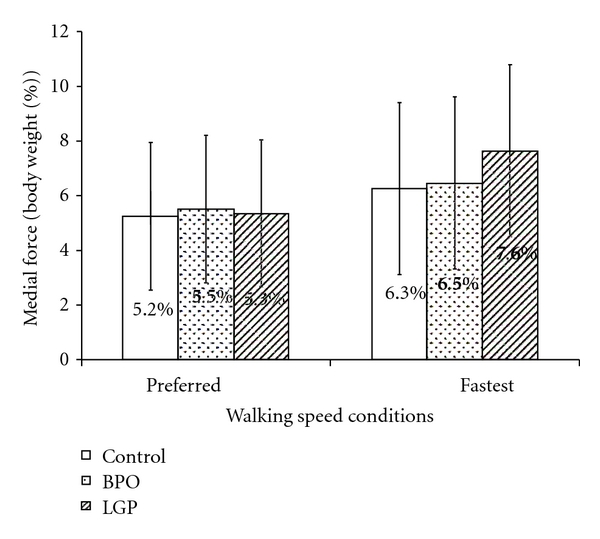
Comparison of peak medial force (percent of body weight). When gait velocity was controlled as a covariate, there was no significant difference in peak medial force among the three groups during both the preferred (*P* = 0.382) and fastest walking speed conditions (*P* = 0.951).

**Figure 2:**
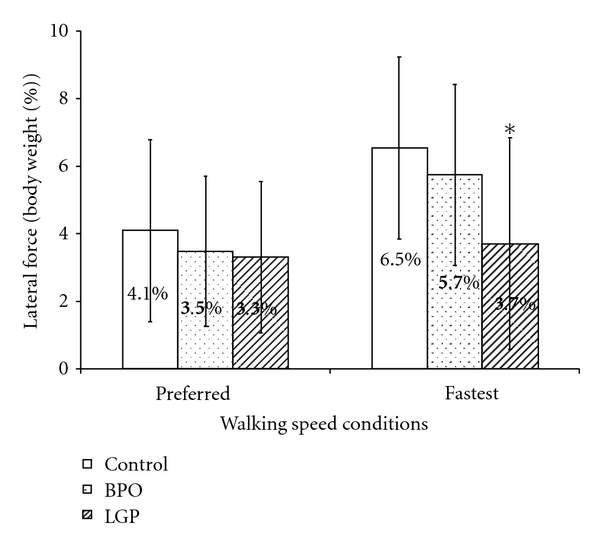
Comparison of peak lateral force (percent of body weight). *When gait velocity was controlled as a covariate, the peak lateral force was significantly less in the LGP group compared to the control group during the fastest walking speed condition (*P* = 0.005). There was no significant difference in peak lateral force among the three groups during preferred speed condition (*P* = 0.619), or between BPO and control groups during fastest walking speed condition (*P* = 0.359).

Peak medial and lateral forces exerted during walking at preferred and fastest speeds and for the three groups are illustrated in Figures 1 and 2. At preferred walking speed and with normalized gait velocity, the peak medial and lateral forces were similar across groups. Specifically, mean peak medial forces were 5.2%, 5.3%, and 5.5% of body weight for control, BPO, and LGP groups, respectively. Peak lateral forces were 4.1%, 3.5%, and 3.3% of body weight for control, BPO, and LGP groups, respectively. At fastest walking speed and with normalized gait velocity, there was a general increase in peak medial and lateral forces for all groups. Specifically, mean peak medial forces were 6.3%, 6.5%, and 7.6% of body weight for control, BPO, and LGP groups, respectively. Peak lateral forces increased and reached were different between groups and were 6.5%, 5.7%, and 3.7% of body weight for control, BPO, and LGP groups, respectively.

**Figure 3:**
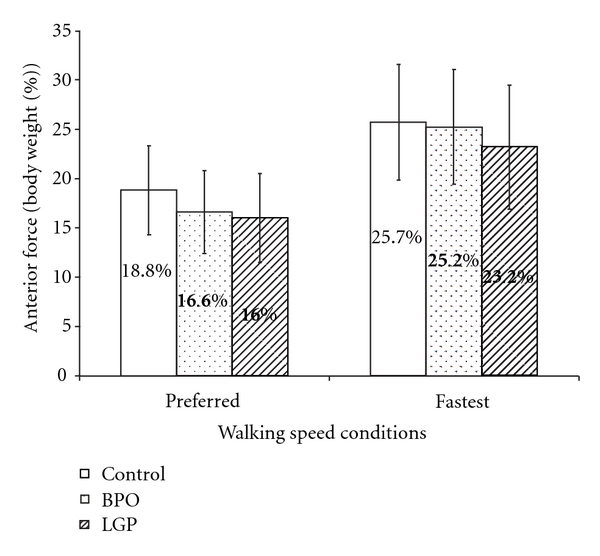
Comparison of peak anterior force (% of body weight). When gait velocity was controlled as a covariate, there was no significant difference in the peak anterior force among the three groups during the preferred (*P* = 0.172) and fastest walking speed conditions (*P* = 0.423).

**Figure 4:**
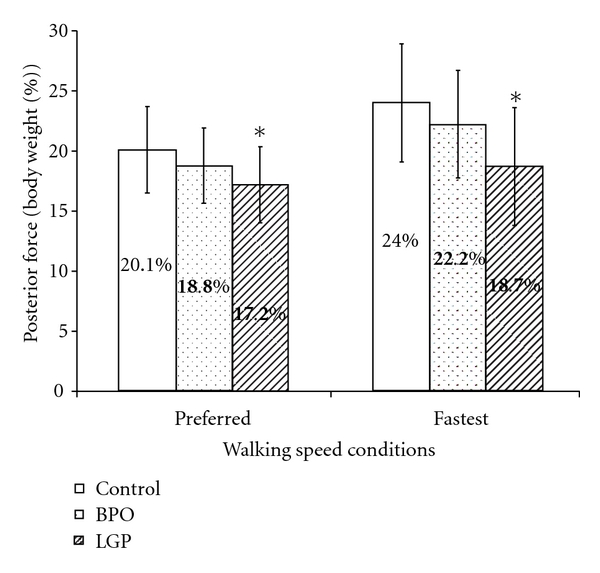
Comparison of peak posterior force (% of body weight). *When gait velocity was controlled as a covariate, the peak posterior force was significantly less in the LGP group compared to the control group during both the preferred walking speed condition and (*P* = 0.013) and the fastest walking speed condition (*P* = 0.002). There was no significant difference in peak posterior force between BPO and control groups during the preferred (*P* = 0.219) and fastest walking speed conditions (*P* = 0.210).

Peak anterior and posterior forces exerted during walking at preferred and fastest speeds and for the three groups are illustrated in Figures 3 and 4. At preferred walking speed and with normalized gait velocity, the peak anterior forces were similar across groups. Specifically, mean peak anterior forces were 18.8%, 16.6%, and 16.0% of body weight for control, BPO, and LGP groups, respectively. Peak posterior forces differed across groups and were 20.1%, 18.8%, and 17.23% of body weight for control, BPO, and LGP groups, respectively. The LGP exerted significantly less posterior (push off) force. At fastest walking speed and with normalized gait velocity, again there was a general increase in the magnitude of peak anterior and posterior forces for all groups. Specifically, mean peak anterior forces were similar across groups and were 25.7%, 25.2%, and 23.2% of body weight for control, BPO, and LGP groups, respectively. Peak posterior forces again increased and were different between groups and were 24.0%, 22.2%, and 18.7% of body weight for control, BPO, and LGP groups, respectively. Again, the LGP exerted significantly less posterior (push off) force.

Correlations among pain, disability, and gait velocity are presented in Table 4. As expected correlations among pain reports of pain intensity and pain affect were strong and significant (range *r* = .49 to *r* = .93). The strongest association was between leg pain affect and intensity and the weakest was between back pain intensity and leg pain affect. Relationships between pain and disability measures were weak to moderate (range *r* = .11 to *r* = .33) and between pain and gait velocity were generally moderate (*r* = .25 to *r* = .46). Finally of the associations between self-report questionnaires (other than pain) and gait velocity, the distorted ambulation scale had the strongest association (*r* = −.44 and *r* = −.46, at fastest and preferred speed, resp.).

Stepwise linear regression analysis showed that significant predictors of gait velocity at preferred speed were back pain intensity (*R* = .46, *R* ^2^ = .21, *F*_1,38_ = 10.26, *P* < .005), and the distorted ambulation scale which together accounted for 31% of the variance (*R* = .56, *R* ^2^ = .31, *F*_2,37_ = 8.26, *P* < .001). The significant predictors of gait velocity at fastest speed were leg pain affect (*R* = .46, *R* ^2^ = .21, *F*_1,38_ = 10.37, *P* < .005), and the distorted ambulation scale which together accounted for 32% of the variance (*R* = .57, *R* ^2^ = .32, *F*_2,37_ = 8.72, *P* < .001).

## 4. Discussion

Walking is a fundamental component of function and is known to be compromised by LBP. This study confirms that specific spatial characteristics of pain differentially influence the extent of that compromise and suggest that walking as an intervention, may be differentially effective, at least in the short term, based on pain distribution.

The self-reported long duration of LBP, including the current episode of LBP is worthy of comment. All subjects with LBP were recruited from an outpatient orthopedic spine clinic in a large urban city and the vast majority reported having a long history of LBP. The mean (9.5 years and 6 years) and maximum duration (33 years and 15 years, in the BPO and LGP groups, resp.), certainly support the notion that LBP is a problem that is not cured but managed. An increased focus on self-management and long-term followups for clinical trials are in order, such that interventions are appropriately tested for effectiveness. Over the last couple of decades, a substantial body of research has focused on testing the efficacy of specific interventions for acute, subacute, and chronic LBP based on identification of the putative structural source of the pain. Whilst this approach has contributed to the cost of treating LBP, it has clearly had a minimal impact on resolving the impact of the problem of LBP.

It was more surprising to see that the length of the current episode was also quite long (i.e., mean duration of one to one and a half years). Although a few people reported a significant reinjury or event that led to their current episode, many reported a gradual increase in the frequency of acute episodes and/or a change in intensity or distribution of pain. The exact and actual duration of the current episode was thus difficult to determine with any level of accuracy and as such should be interpreted with caution. That said, the patient's perception of the duration is clear and may reflect their belief that their LBP seems like an interminable problem.

In regards to walking it was interesting to note that for the BPO group walking had an analgesic effect—this was not the case for the LGP group. It is not clear whether this analgesic effect would persist with walks of longer duration and or at a sustained fast speed. However, the differential results across groups do suggest that pain distribution should be considered when clinicians make recommendations for walking. And communications to the patient regarding the expected benefits of walking should probably be tailored accordingly.

Pain distribution had a significant impact on walking velocity and horizontal ground reaction forces, and this impact became greater across groups as the physical challenge increased, that is, at fast walking speeds. It was not surprising therefore to find that pain intensity (BPO) and pain affect (LGP) predicted velocity at both preferred and fastest speeds and the additional predictive value of the DAS is corroborative.

### 4.1. Comparison of Peak Mediolateral Forces

The results support the hypothesis that the BPO and control groups would generate similar peak medial and lateral forces under both walking speed conditions, with gait velocity controlled as a covariate. In contrast, the hypothesis that the LGP group would develop significantly less peak lateral force at both walking speeds was only partially verified. Peak lateral force in the LGP group was significantly less than that of the other two groups but only at the fastest walking speed. The peak lateral force at the preferred walking speed condition and the peak medial force at both walking speeds were similar between the LGP and control and BPO groups.

It is reasonable that both BPO and LGP groups generated peak medial forces that were similar to the medial forces generated by the control group. Only a small change in medial force is possible during gait. Medial force is limited by the positioning of the adducting loading lower limb which does not usually cross over the line of progression during walking unless there is a major balance problem. This was clearly not the case for the subjects in this study.

Peak lateral force comparisons differed depending on pain distribution. Both BPO and control groups generated similar peak lateral forces during preferred and fastest walking speed. In contrast, the LGP group generated significantly less peak lateral force than the control group at their fastest walking speed, but not at their preferred walking speed. This suggests that people with LGP seem to walk with less lateral displacement (i.e., closer to the line of progression) than their pain-free counterpart, particularly when they are challenged to walk at their fastest speed. Considering the LGP group in this study did not experience significant change in pre- posttest pain, the reduction in lateral displacement and may be a protective strategy against potential movements anticipated to aggravate pain. Although, delayed, and, or prolonged trunk muscle response time in people with LBP has been posited as an underlying reason for the reduction in lateral displacement during walking, this is a less likely driver than actual or anticipated pain.

Indeed, postural sway in people with LBP appears to be task-specific. Increased postural sway in individuals with LBP has been demonstrated during stationary standing and sitting tasks and in standing from and sitting. However, this was not evident during the dynamic walking task in this study.

### 4.2. Comparison of Peak Anteroposterior Forces

The results support the hypothesis that both BPO and control groups would generate similar peak anterior and posterior forces under both walking speed conditions. With the exception of peak anterior force, the results also support the hypothesis that LGP group would generate significantly less peak posterior force than the control group under both walking speed conditions. These findings suggest leg pain is an important factor influencing the amount of peak posterior force (i.e., driving force) during walking, but not peak anterior force (i.e., braking force).

It is reasonable that individuals, regardless of pain, generate similar peak anterior force (braking force) when gait velocity is controlled as a covariate. Similar findings have been reported in people with total knee arthroplasty who experienced residual leg pain. Braking force occurs during a very brief time interval (10%) during the initial stance phase. Thus, walking speed is likely the only factor that individuals can control to influence the amount of braking force. Clinicians should, therefore, be mindful of walking speed when recommending walking exercise to people with LBP.

Peak posterior force (driving force) varied depending on the presence of leg pain during both walking speed conditions. Both BPO and control group generated similar driving force, but LGP group had less driving force than the control group despite controlling for gait velocity in both walking speed. Those with total knee arthroplasty also had similar findings to the LGP group in this study [20]. These observations suggest that besides walking slower, those with leg pain also utilized other strategies to actively control the amount of driving force. One such strategy is the taking of shorter contralateral steps [21], which may explain the observed asymmetric gait pattern in those with leg pain [30, 31]. Higher Distorted Ambulation Scale score reported by the LGP group than the BPO group in this study corroborates this suggestion.

### 4.3. Integrated Group Comparisons of Vertical and Horizontal GRFs

Taking together both vertical [8] and horizontal GRF results, people with LGP tend to utilize different gait strategies under different walking speed conditions. When people with LGP have a choice to walk at their preferred speed, they tend to walk slower and generate less posterior resultant forces in both horizontal (driving force) and vertical components (push-off force and push-off rate) than their pain-free counterparts. However, when those with LGP are challenged to walk as fast as they can, they walk slower with less lateral and posterior resultant forces primarily in the horizontal components (driving force and lateral force) than their pain-free counterparts. The long term effects of altered gait strategies on LBP are not clear. Neither is it really clear whether the differences identified are causes, consequences, correlates, or simply interesting observations in this complex problem. Further longitudinal studies are required to address these questions, and also to determine optimal walking recommendations for those with LBP and particularly those with LGP.

## 5. Conclusion

Pain distribution in people with LBP differentially influences walking velocity as well as horizontal GRF generated. People with BPO prefer to walk slower than their pain-free counterparts but can walk faster; those with LGP do not have that capacity. In addition people with BPO walk more “normally” as they generated comparable horizontal GRF as pain-free individuals in both preferred and fastest walking speeds. In contrast, people with LGP walked with less driving force at both preferred and fastest walking speeds, and generated less lateral force at their fastest walking speed than their pain-free counterparts. These results suggest people with LGP utilize significantly altered gait strategies that become more apparent when challenged to walk at faster speeds.

##  Disclosure

The authors certify that no party having a direct interest in the results of the research supporting this paper has or will confer a benefit on them or on any organization with which we are associated AND, if applicable, they certify that all financial and material support for this research and work are clearly identified in the title page of the paper. 

## Figures and Tables

**Table 1:** Demographics of the control, back pain only (BPO) and back pain with unilateral referred leg pain (LGP) groups.

Parameters	Control (*n* = 20) 12 female 8 male	BPO (*n* = 20) 12 female 8 male	LGP (*n* = 20) 12 female 8 male
	Mean ± SD (range)	Mean ± SD (range)	Mean ± SD (range)
Age (yrs)	46.4 ± 11.0 (27–65)	46.0 + 10.6 (25–61)	46.1 ± 10.6 (25–62)
Height (cm)	167.8 ± 9.4 (152.4–182.9)	171.6 ± 11.3 (160.0–190.5)	171.2 ± 9.5 (154.9–188.0)
Weight (kg)	71.7 ± 15.6 (45.8–105.2)	78.6 ± 19.1 (48.9–117.9)	72.8 ± 14.3 (51.4–100.2)
Body mass index (kg/m2)	25.3 ± 4.2 (19.4–35.3)	26.5 ± 5.5 (19.1–40.9)	24.8 ± 4.4 (19.7–33.8)
True leg length discrepancy (cm)	0.8 ± 0.5 (0.0–2.0)	0.6 ± 0.5 (0.0–1.5)	0.7 ± 0.5 (0.0–2.0)

**Table 2:** Descriptive statistics of the self-report questionnaires.

Parameters	BPO^a^ (*n* = 20)		LGP^b^ (*n* = 20)	

Pain duration	Mean ± SD (range)		Mean ± SD (range)	

1st episode (months)	112.0 ± 110.7 (12.0–396.0)		73.7 ± 58.4 (1.0–180.0)	
Present episode (months)	17.9 ± 32.3 (0.2–120.0)		11.9 ± 14.0 (1.0–60.0)	

Pretest pain distribution (based on pain drawing diagram)				

Above gluteal fold	20		20	
Between gluteal fold and knee	0		6	
Below knee	0		14	

VAS^c^	Mean ± SD (range)		Mean ± SD (range)	
	Pretest	Posttest	Pretest	Posttest
Back pain intensity (cm)	3.9 ± 2.1 (0.8–7.9)	2.9 ± 2.4* (0–7.8)	5.1 ± 2.2 (1.2–8.3)	5.5 ± 2.7 (0–8.3)

Back pain affect (cm)	4.4 ± 2.4 (0.4–10.0)	3.2 ± 2.3* (0–7.2)	6.0 ± 2.4 (0.6–9.6)	5.5 ± 2.9 (0–8.6)
Examined leg pain intensity (cm)	—	—	5.0 ± 2.2 (1.4–7.6)	4.9 ± 2.1 (1.9–8.0)
Examined leg pain affect (cm)	—	—	5.5 ± 2.6 (1.0–9.6)	5.0 ± 2.3 (1.1–8.4)

	Mean ± SD (range)		Mean ± SD (range)	

RMDQ^d^ (0–24)	9.5 ± 5.9 (0–20)		12.5 ± 4.3 (4–21)	
FABQ-Walk^e^ (0–30)	6.5 ± 6.0 (0–19)		10.6 ± 7.4 (0–23)	
DAS^f^ (0–36)	9.6 ± 7.8 (0–30)		12.9 ± 7.0 (0–25)	

*BPO group had significantly less back pain intensity and affect at post -test than at Pretest (*P* = 0.04).

^a^BPO: back pain only group.

^b^LGP: back pain with referred leg pain group.

^c^VAS: Visual Analogue Scale.

^d^RMDQ: Roland and Morris disability questionnaire.

^e^FABQ-Walk: Fear Avoidance Belief Questionnaire—physical activity section (emphasis on walking).

^f^DAS: Pain Behavior Check List—Distorted Ambulation Scale.

**Table 3:** Mean and standard deviation of absolute and relative gait velocity for the control, back pain only (BPO) and back pain with unilateral referred leg pain (L GP) groups.

Walking speed conditions	Relative gait velocity	Control	BPO	LGP
		Mean ± SD	Mean ± SD	Mean ± SD
Preferred	Normalized (% body height per second)	0.9 ± 0.1	0.8 ± 0.9*	0.8 ± 0.1†
	Absolute (m/s)	1.6 ± 0.5	1.3 ± 0.5	1.3 ± 0.5
Fastest	Normalized (% body height per second)	1.3 ± 0.1	1.2 ± 0.2	1.0 ± 0.2†
	Absolute (m/s)	2.1 ± 0.8	2.1 ± 0.8	1.7 ± 0.8

*The BPO group walked significantly slower than the control group during the preferred walking speed condition (*P* < .0005).

†The LGP group walked significantly slower than the control group in both the preferred and fastest walking speed conditions (*P* = .002).

**Table 4:** Pearson's correlation coefficients among pain, disability, and gait velocity (*n* = 40).

Variable	Back pain intensity	Back pain affect	Leg pain intensity	Leg pain affect	Roland and Morris	Fear-avoidance beliefs	Distorted ambulation	Preferred gait velocity	Fast gait velocity
Back Pain Intensity	1	.756**	.540**	.490**	.111	.131	.078	−.345*	−.308*
Back Pain Affect		1	.516**	.600**	.168	.138	.116	−.324*	−.219
Leg Pain Intensity			1	934**	.272	.231	.327*	−.249	−.437**
Leg Pain Affect				1	.248	.212	.270	−.297	−.463
Roland and Morris					1	.361*	.698**	−.274	−.332*
Fear Avoidance Beliefs						1	.441**	−.117	−.294
Distorted Ambulation							1	−.461**	−.438**
Preferred Gait Speed								1	.695**
Fast Gait Speed									1

**P < 0.01, *P < 0.05.
